# Long-term experimental hybridisation results in the evolution of a new sex chromosome in swordtail fish

**DOI:** 10.1038/s41467-018-07648-2

**Published:** 2018-12-03

**Authors:** Paolo Franchini, Julia C. Jones, Peiwen Xiong, Susanne Kneitz, Zachariah Gompert, Wesley C. Warren, Ronald B. Walter, Axel Meyer, Manfred Schartl

**Affiliations:** 1 Lehrstuhl für Zoologie und Evolutionsbiologie, Department of Biology, University of Konstanz, Universitätsstraße 10, 78457 Konstanz, Germany; 2 Department of Medical Biochemistry and Microbiology, Uppsala University, Uppsala, 75123 Sweden; 3 Physiological Chemistry, Biozentrum, University of Würzburg, Am Hubland, 97074 Würzburg, Germany; 4 Department of Biology, Utah State University, Logan, UT 84322 USA; 5 McDonnell Genome Institute, Washington University School of Medicine, St. Louis, 63108 MO USA; 6 The Xiphophorus Genetic Stock Center, Department of Chemistry and Biochemistry, Texas State University, San Marcos, 78666-4616 TX USA; 7 Radcliffe Institute for Advanced Study, Harvard University, 9 Garden Street, Cambridge, MA 02139 USA; 8 Comprehensive Cancer Centre, University Clinic Würzburg, Josef Schneider Straße 6, 97074 Würzburg, Germany; 9 Hagler Institute for Advanced Study and Department of Biology, Texas A&M University, College Station, TX 77843 USA

## Abstract

The remarkable diversity of sex determination mechanisms known in fish may be fuelled by exceptionally high rates of sex chromosome turnovers or transitions. However, the evolutionary causes and genomic mechanisms underlying this variation and instability are yet to be understood. Here we report on an over 30-year evolutionary experiment in which we tested the genomic consequences of hybridisation and selection between two *Xiphophorus* fish species with different sex chromosome systems. We find that introgression and imposing selection for pigmentation phenotypes results in the retention of an unexpectedly large maternally derived genomic region. During the hybridisation process, the sex-determining region of the X chromosome from one parental species was translocated to an autosome in the hybrids leading to the evolution of a new sex chromosome. Our results highlight the complexity of factors contributing to patterns observed in hybrid genomes, and we experimentally demonstrate that hybridisation can catalyze rapid evolution of a new sex chromosome.

## Abstract (web summary)

Fish have a high diversity of sex-determining systems, but the mechanisms responsible for this are not well understood. Here, Franchini et al. show how hybridization and backcrossing have led to the evolution of a new sex chromosome in swordtail fish during 30 years of experimental evolution.

## Introduction

Sex chromosomes evolve from autosomes and are typically extremely conserved, not only among species but entire classes of organisms. The best-studied sex chromosome systems are ZW and XY, female and male heterogametic systems respectively. These categorical sex-determining systems have evolved repeatedly and independently in the animal and plant kingdoms^1^. Yet genomic studies have also revealed several examples of rapidly evolving sex-determining mechanisms in closely related species of fish^2–7^, and also in amphibians^8,9^ and reptiles^10,11^. Even between sister taxa of fishes, instances are known where either males or females are heterogametic. However, despite much theoretical work and empirical findings of convergent evolution of sex chromosomes^11–16^, why such variety in something as fundamental as the mechanisms of sex determination exists, and how it evolves, remains elusive.

If two closely related lineages differ in their sex chromosome systems, several scenarios for the emergence of this difference are possible^17^: either there was no chromosomal sex determination in the last common ancestor and both systems developed independently, or one lineage retained the ancestral sex chromosomes while in the other lineage a transition to the alternate system occurred. Theoretically, such transitions can happen on the same pair of sex chromosomes (homologous transition) or involve an autosome, which then becomes a new sex chromosome (heterologous transition). At the molecular level, the gene(s) determining sex might have changed their mode of action, e.g. from female to male determination, or a novel sex-determining (SD) gene might have arisen and taken over (SD turnover) in the evolving lineage.

An intriguing scenario in sex chromosome evolution connected to speciation is hybridisation, particularly in crosses of species with different male and female heterogametic sex chromosomes. Based on Haldane’s rule, one would expect that the heterogametic sex in hybrids tends to show greater inviability or sterility than the homogametic sex^18^ and one proposed explanation for this is dominance theory^19^. Hybrids between different species might be expected to suffer deleterious incompatibilities because alleles from orthologous genes from different species may not interact well in hybrids, and if these genes are on the sex chromosomes and are recessive, the heterogametic sex is likely to suffer the most. This would be expected to result in selection for alternative sex chromosome systems in lineages arising from a hybridisation event. As intriguing as these concepts are, empirical evidence for the evolution of new sex chromosome systems following hybridization is very scant because these events have happened in the evolutionary past and would be expected to take long time periods to complete. Still, in some laboratory animals the loss of a sex-determining locus has been observed (e.g. medaka^20^ and zebrafish^21^), but these domesticated lines have not yet developed a new stable mechanism of the same kind (a sex determination turnover) or completed a transition.

In vertebrates, teleost fish show an enormous diversity of sex chromosome systems, hence turnovers or transitions must have occurred more often than in most other classes of vertebrates (e.g. ref. ^7^). Therefore, the study of closely related species of fishes that exhibit different sex chromosome systems would be particularly informative for the investigation of the evolution of mechanisms of sex determination (e.g. ref. ^7^). Among the 26 species of the genus *Xiphophorus* (swordtails and platyfish) are species with both XY and ZW sex chromosome systems^22,23^. In this genus, these simple heterogametic systems are both present together with more complex situations that can include multiple loci and chromosomes^23,24^. *Xiphophorus maculatus*, for example, one of the most well studied and geographically widespread species in this genus, has three different genetically well-defined sex chromosomes, X, Y and W. Possible female genotypes are XX, XW and YW, and in males XY or YY. Several different models have been proposed to explain sex determination with three sex chromosomes in this species^25,26^. While in the wild in most populations all these sex chromosome combinations co-exist, laboratory lines have been established which are stable for XY/XX or WY/YY sex determination. WW females can be generated in the laboratory after experimental manipulation and are viable, but such individuals have not been reported in natural populations^23^. In several species, autosomal modifiers, which occur at low frequency in natural populations, have also been reported to act, and may explain instances of atypical sex determination^24,25^. As another example of sex determination variation in this genus, in *Xiphophorus* hellerii, a polyfactorial sex-determining system has been reported to be acting. Additionally, it has been suggested that this species has a main genetic system affected by numerous autosomal modifiers, while more recent studies report that some strains of *X. hellerii* have a XW–YY female heterogametic system^22,23^. To our knowledge, environmental sex determination has not been reported from observations in nature or from laboratory studies in the genus *Xiphophorus*.

In general, this genus of fish comprises well known and widely studied models for organismic and molecular evolution. In particular, *Xiphophorus* species have been used as a model system for studying the evolutionary genetics of hybridisation for more than 50 years^27–32^ with a recent resurgence of investigations using modern molecular and genomic data^27,33–38^. Ancient hybridisation events between many species in this genus has been inferred^27,36,37,39^ and may be facilitated by apparently weak postzygotic isolation in *Xiphophorus*^39–41^. We previously utilized SNP data from RAD sequencing to estimate the phylogenetic relationships among all 26 *Xiphophorus* species, and uncovered incongruence between the RAD-nuclear vs. mtDNA phylogenies^33,36,42^. This phylogenetic incongruence likely reflects the contribution of hybridisation to the evolution of two *Xiphophorus* species (*Xiphophorus* clemenciae and *Xiphophorus* monticolus)^36^. Such phylogenetic incongruence, in addition to asymmetrical behavioural preferences of first generation hybrids and the morphology of the putative hybrid taxon being much closer to one of the possible parental species^33^, provides support for hybridisation followed by repeated backcrossing having contributed to the evolution of these species. The inferred ancient hybridisation event likely involved a female Southern platyfish, *X. maculatus*, and a male green swordtail, *X. hellerii*.

**Fig. 1:**
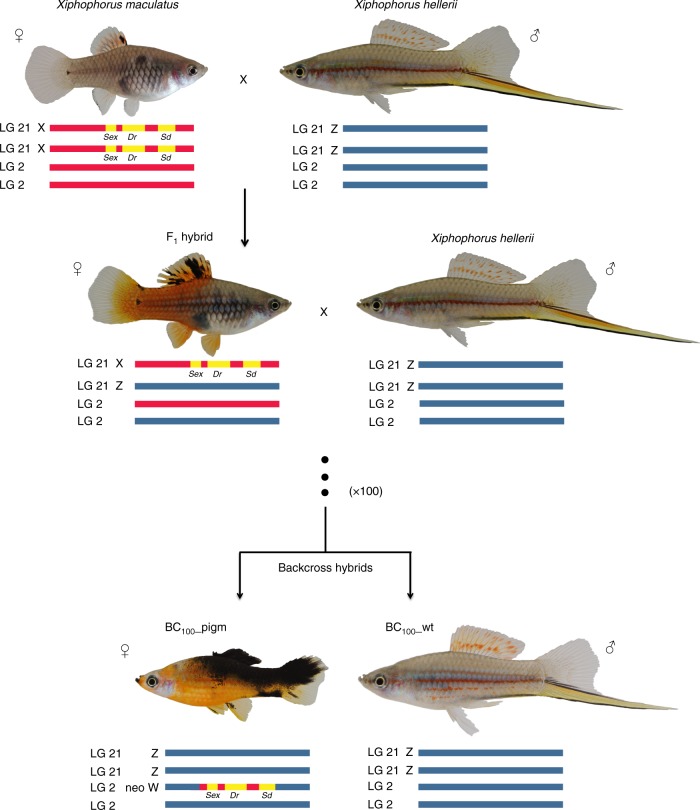
Schematic outline of the laboratory cross. The parental species *X. maculatus* and *X. hellerii*, a female specimen of the first generation hybrid and the resulting offspring obtained after approximately 100 generation of backcrosses (see Methods for details) are shown. The *dorsal red* (*Dr*), the *spotted dorsal* (*Sd*) and the oncogene *xmrk* loci, closely linked on the X-chromosome of the platyfish (LG21), are also highlighted. A translocation event of a genomic region containing these loci accompanied by recombination suppression has likely contributed to the formation of a new sex chromosome, a neo W chromosome (LG2), during the backcross experiment. Fish images by M. Schartl

Evidence for reticulate evolution is typically inferred from analyses of extant populations or species. However, the actual processes contributing to the introduction of an allospecific genome and the fate of the extra-specific genetic material are difficult to identify and document. To better understand the genomic consequences of interspecific hybridisation for sex chromosome evolution and speciation, we conducted a long-term crossing experiment (conducted for >30 years and spanning >100 generations) in fish of the genus *Xiphophorus* (Fig. 1). We experimentally mimic the evolutionary scenario, hybridisation with repeated backcrossing, which is thought to have contributed to the evolution of at least two of the 26 species in the genus *Xiphophorus*^33,36,37^. Of particular interest is that the parental species used in the experimental evolutionary crosses differ in their sex chromosome systems. The maternal lineage used here, the platyfish *X. maculatus* Jp163A strain, has an XY sex chromosome system^43^, whereas the backcross paternal lineage, the swordtail *X. hellerii* strain originating in the Rio Lancetilla, has a ZW sex chromosome system. This experimental set up enabled us to investigate the genomic consequences of *known* hybrid ancestry and timing, and specifically the consequences of sex chromosome evolution. We find that during the process of continuous introgressive breeding, the sex-determining region of the X chromosome derived from the maternal ancestor was translocated to an autosome of the receiving genome, thus providing empirical experimental evidence of a heterologous transition. We also note that at its new chromosomal position the former X-chromosome-specific region is located in a large region of retained maternal sequences, indicating that the loss of recombination accompanied the establishment of the novel W chromosome.

## Results

### Identification of the sex-determining system and sex linkage group in *X. hellerii*

The sex determination system of the platyfish strain used for our study is firmly established as XY, not only from a plethora of crossing data but also from molecular genetic evidence, and there is no evidence for an influence of autosomal or environmental modifiers (e.g. the platyfish genome was based on the same strain^43^). However, for *X. hellerii* the situation is less clear and there are conflicting reports ranging from pure ZW heterogamety to a polyfactorial system with the absence of sex chromosomes^44,45^. To identify the sex-determining (SD) system in *X. hellerii*, deep coverage RAD sequencing of 60 *X. hellerii* individuals (30 males and 30 females) of the Rio Lancetilla strain was done (sequencing statistics for each individual is provided in Supplementary Data 1).

**Fig. 2:**
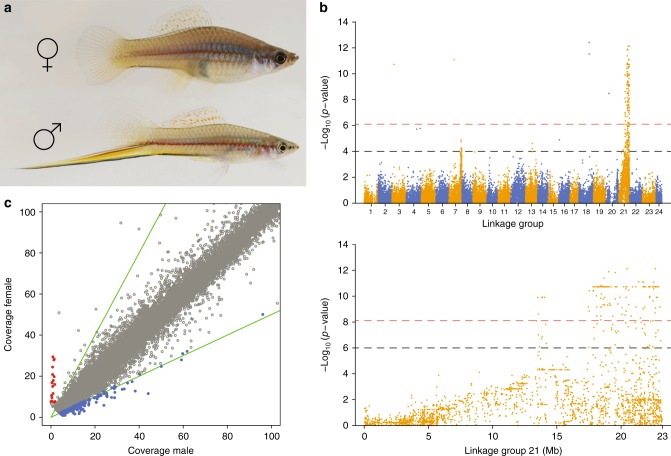
Sex-determination system in *Xiphophorus hellerii*. **a** A male and female specimen of *X. hellerii*, males are clearly distinguished by the elongated caudal fin, ‘the sword’ (Fish images by M. Schartl). **b** Genome-wide association analysis, where sex is set as a binary case/control variable. The 65,417 SNPs that passed quality control were used in this analysis. The Manhattan plot shows the −log_10_ *p*-value (Fisher’s exact test) of each SNP across the 24 *X. maculatus* linkage groups (LGs). The dashed red line indicates the genome-wide significance level (*p*-value = 7.79e^−7^), while a suggestive significance (*p*-value = 0.001) is denoted by the dashed black line. Orange and blue colours are used to distinguish between LGs. The peak showing the strongest association is located on LG21, and is enlarged in the bottom-right section of the panel. **c** Sequence coverage of RAD loci in female and male *X. hellerii* individuals. Each dot in the scatter plot represents the average coverage across all males (*x*-axis) against the average coverage across all females (*y*-axis) for each of the selected RAD loci. Red dots indicate potential W-linked loci (female-specific loci), while blue dots indicate loci potentially linked to the Z chromosome (twice the coverage in males than in females). The upper and lower green lines show the expected coverage of X-linked and Z-linked loci in a XY and ZW sex determination system, respectively. The coverage plot showing the total number of selected loci (average coverage across all individuals >3 and <400) is reported in Supplementary Fig. 2

To identify genomic regions associated with sex in *X. hellerii*, we employed a genome-wide association study (GWAS) and an analysis of genetic differentiation (F_ST_). For these analyses, we used the *X. maculatus* v4.4.2 as the reference genome^46^, where the sex chromosome pair was clearly detected in linkage group 21 (LG21). Given the high level of synteny conservation between the two species’ genomes^47^ this approach allowed us to directly compare the sex chromosome regions of *X. maculatus* and *X. hellerii* by plotting the GWAS *p*-values and the F_ST_ estimates on the *X. maculatus* linkage groups. The GWAS approach identified a large genomic region strongly associated with sex distal on LG21, spanning more than 1/3 of the LG (a genomic region of approximately 10 Mb, from ~13 to ~23 Mb, at the end of LG21) (Fig. 2b). This region includes 243 SNPs that exceeded the Bonferroni-corrected genome-wide threshold (*p*-value = 7.64e^−7^) and showed an excess of heterozygosity in females vs. males, conforming to expectations under a ZW sex determination system. A weaker association was identified in LG7, where 15 SNPs exceeded a threshold suggestive of significance (*p* < 0.0001) (the location of the SNPs highly associated with sex that exceed the thresholds are shown in Supplementary Data 2). A second region of increased female sex linked heterozygosity besides LG21 representing the W/Z pair was unexpected. A reasonable explanation is that this region may play a role as a minor sex modifier. Such autosomal modifiers of a major sex chromosomal system have frequently been noted in the genus *Xiphophorus*^24^. Further, in our laboratory cross experiments (see below) we observed exceptional cases of males with a BC_100__pigm phenotype and females with a BC_100__wt phenotype (Supplementary Table 1). Such cases may be caused by autosomal modifiers.

F_ST_ analyses confirmed our findings using the GWAS approach. Several genomic regions exhibited a high degree of genetic differentiation between males and females on LG21 (F_ST_ > 0.5) on a background of low genome-wide differentiation (average F_ST_ across all loci = 0.004). This pattern was consistent across the three different sliding windows used (1, 10 and 100 Kb). F_ST_ analysis confirmed the weaker association found on LG7 using the GWAS approach. Across the three windows used, several regions consistently exceeded the set threshold, showing an average F_ST_ value in the upper 1% of the distribution of the per window F_ST_ values. However, the majority of the windows exceeding the threshold were located on LG21 (see Supplementary Data 3 and Supplementary Fig. 1).

To provide further confirmation of the genomic regions associated with sex in *X. hellerii*, we analysed coverage information from males and females in order to identify any non-recombinant regions along chromosomes. Here, we used the *X. hellerii* genome v3.0.1^47^ for aligning the RAD reads, derived from a single female, the heterogametic sex in a W/Z sex chromosome system. By investigating copy number variation, we detected 18 candidate female-specific loci (mean coverage in females >7; mean coverage in males <2) (Fig. 2c, Supplementary Fig. 2 and Supplementary Data 4) and no male-specific loci. Again, such a pattern is compatible with female heterogamety and a W/Z sex chromosome system. We then aligned these loci to the *X. maculatus* genome v4.4.2 to compare the location of the sex-determination system in the two *Xiphophorus* species. We found that out of the total 18 female-specific loci, 16 aligned to the *X. maculatus* genome, 14 of which were on LG21, which is the sex linkage group (Supplementary Data 4). This indicates that the sex-chromosome pairs of *X. hellerii* and *X. maculatus* are homologous. We then identified potential Z-linked loci (mean coverage ratio in male/female >1.9) (Fig. 2c, Supplementary Fig. 2 and Supplementary Data 4). Of these, we found that 161 loci aligned to the *X. maculatus* genome, and the highest number of loci aligned to LG21 (54: 33.5%), followed by LG13 (27: 16.8%) and LG10 (17: 10.5%) (Supplementary Fig. 3 and Supplementary Data 4). Both the W-linked female-specific loci and the Z-linked male loci were found to be over-represented on LG21 (two-tailed Fisher exact test: *p*-value < 0.001). The occurrence of several Z-linked loci that did not align to *X. maculatus* LG21 could suggest the presence of autosomal modifiers. However, given the potentially high false positive rate when using such an approach, caution is needed in interpreting these findings.

Taken together the GWAS, F_ST_ and coverage analysis results show that the Rio Lancetilla population of *X. hellerii*, which is the paternal species in our long-term crossing experiment, has a ZW chromosome system. LG21 is the sex chromosome pair and includes a terminal non-recombining region (spanning from approximately 13 to 23 Mb) and a large (0–13 Mb) pseudoautosomal region (PAR). The boundary between the non-recombining region and the PARs was identified by a sudden drop of SNPs significantly associated with sex, as revealed by the GWAS (Fig. 2b and Supplementary Data 2), a pattern confirmed by the F_ST_ (Supplementary Fig. 1 and Supplementary Data 3) and the coverage analysis (Supplementary Data 4). Despite *X. maculatus* having a different type of genetic sex determination, the sex chromosomes of both parental species are homologous.

### Introgression of parental DNA in a controlled backcrossing experiment

We performed a laboratory cross, hybridisation with backcrossing, and sequenced the first and approximately 100th generation backcross individuals (sequencing statistics for each individual are provided in Supplementary Data 5). This crossing experiment mimicked the evolutionary scenario that has potentially given rise to two *Xiphophorus* fish species, *X. clemenciae* and *X. monticolus*, and in which the parental species differ in their sex chromosome systems (see refs. ^26,33,36,37,43^). Accordingly, we crossed a female Southern platyfish (*X. maculatus*, genotype 2*N* = 48; XX) with a male swordtail (*X. hellerii*, genotype 2*N* = 48; ZZ). A female F_1_ fish was used for producing the first backcross with *X. hellerii*. Further on, female fish were selected in each backcross generation for two pigmentation phenotypes that are encoded on *X. maculatus* X-chromosome (LG21) close to a molecular marker, the *xmrk* oncogene, and mated to *X. hellerii* males.

Not unexpectedly, the crossing of two species with opposing sex chromosomal systems produced an F_1_ generation that showed severe signs of hybrid dysgenesis. In such crossing, the number of offspring that developed to adulthood was very low (usually 1–3, compared to 20–50 in the purebred parental species) and a considerable number of these were sterile (Supplementary Table 1). This was particularly the case for females, which carry the *X. maculatus* X chromosome, which was our target for selection in the long-term crossing experiment. Conversely, those offspring from backcrosses to *X. hellerii*, including BC_100_, displayed full fertility and a clear-cut sex-linked inheritance of the pigmentation loci (Supplementary Table 2). All fish (99.7%) with these markers were females, while the fish without the selected markers were preponderantly males (91.7%).

**Fig. 3:**
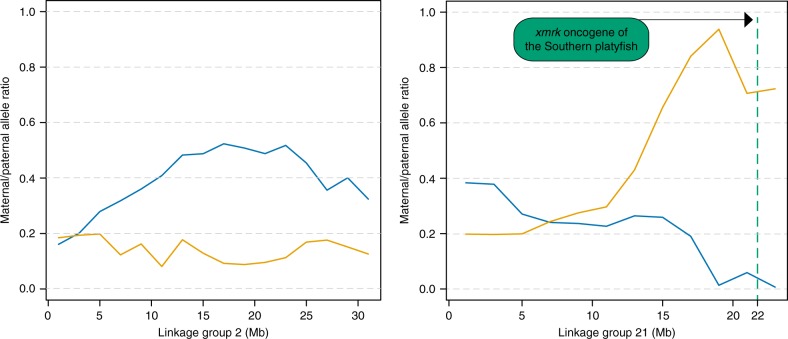
Parental allele ratios in the first generation backcross. Maternal and paternal allele ratios in BC_1__wt (blue line) and BC_1__pigm (orange line) on LG2 and LG21. The location of the melanoma gene (*Xmrk*), linked to the pigmentation genes, in LG21 is shown

**Fig. 4:**
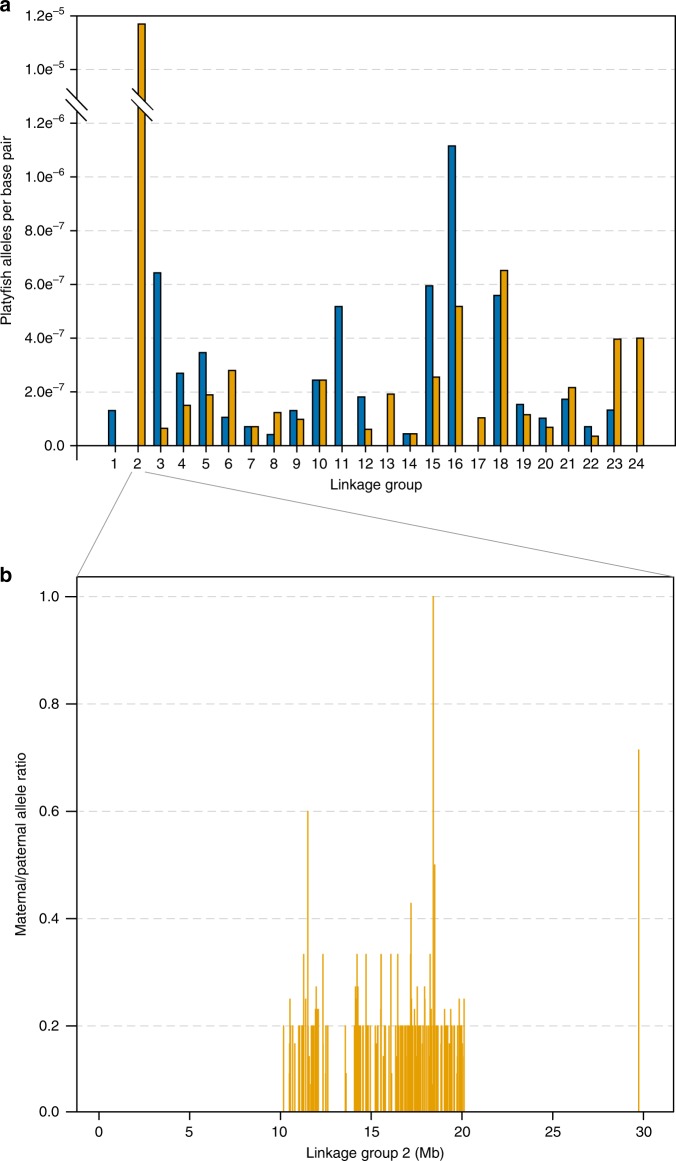
Segregation patterns of parental alleles. **a** Frequency of loci carrying platyfish alleles in the BC_100__wt (blue bars) and BC_100__pigm (orange bars) in each of the 24 linkage groups of the *X. maculatus* genome; **b** frequency of maternal alleles across linkage group 2 (LG2)

A clear enrichment of maternal alleles was observed in the BC_1__pigm group (Fig. 3) as expected at LG21 (maternal/paternal allele ratio >0.9) in a genomic region close to the *xmrk* oncogene of *X. maculatus* (Fig. 3). In the >100 backcross generation, maternal polymorphisms were found at 476 SNPs in the BC_100__pigm group, and at 158 SNPs in the BC_100__wt group (Table 1 and Fig. 4; see the ancestry frequency calculated at each SNP in Supplementary Fig. 4). Notably, in the BC_100__pigm a large block of retained maternal polymorphisms (204 SNPs) spanning approximately 10 Mb was found in the central part of LG2 (Fig. 4). Specifically, several loci with *X. maculatus* alleles were retained in the BC_100__pigm group, which in the genome of the parental *X. maculatus* exclusively identify scaffolds from LG21 that are located in the region containing *xmrk* and the pigmentation loci. No such enrichment was seen on LG21 of the BC_100__pigm group. Hence, this genomic region in the backcross hybrids is derived from the *X. hellerii* genome.

**Table 1:** Segregation patterns of the parental alleles

Group	Class	Paternal alleles (*X. hellerii*)	Maternal alleles (*X. maculatus*)
F_1_	Observed^a^	130,379 (0.500)	130,379 (0.500)
BC_1__wt	Observed	263,923 (0.743)	91,329 (0.257)
	Expected	266,439 (0.750)	88,813 (0.250)
BC_1__pigm	Observed	336,355 (0.771)	100,059 (0.229)
	Expected	327,310 (0.750)	109,103 (0.250)
BC_100__wt	Observed	525,244 (~1.000)	258 (158 sites)
	Expected	525,502 (~1.000)	~0 (~0.000)
BC_100__pigm	Observed	513,445 (~1.000)	1073 (476 sites)
	Expected	514,518 (~1.000)	~0 (~0.000)

For each group, the observed and expected number of alleles and their proportions (in parentheses) are shown

^a^Observed and expected number of alleles have the same values for the F_1_ class as a result of the quality filter we applied to the loci selection

To identify genomic regions in hybrid individuals that harbour *X. maculatus* polymorphisms, parental alleles were sorted according to a set of key criteria, sites carrying a maternal variant in at least one individual of the BC_100_ generation were identified and annotated, and the ancestry frequency of each SNP was calculated. For this analysis, the region in the BC_100__pigm showing high maternal allele density in LG2 was masked because of the hypothesized different mechanisms by which it was produced (translocation of the *X. maculatus* sex-determining region to an autosome of the receiving genome). After removing these sites, a total of 204 loci with maternal alleles were identified in the BC_100._ Our simulations show that the number of retained *X. maculatus* polymorphisms is higher than expected under a neutral model of hybridisation with backcrossing (*p* < 0.001; Fig. 4). This suggests that selection has shaped the hybrid genomes beyond LG2.

## Discussion

By conducting a long-term evolutionary crossing experiment, we demonstrate the genomic consequences, and particularly the consequences of sex chromosome evolution, of hybridisation with backcrossing to the paternal species. This same mechanism has potentially contributed to the evolutionary history of two *Xiphophorus* fish species. Importantly, the parental species in this cross differ in their sex chromosome systems. We find that artificial selection for pigmentation phenotypes in backcrossed individuals results in the retention of a large, maternally derived genomic block after 100 generations (>30 years). Interestingly, this experimental design has unexpectedly promoted the formation of a new sex chromosome, and although no other known selection is imposed under our experimental conditions, there is also a higher proportion of maternal SNPs retained elsewhere in the genome than expected under a neutral model. The retention of certain *X. maculatus* alleles in synthetic swordtail–platyfish hybrids thus appears to be a phenomenon that occurs across a very short timescale and may be driven by a range of non-mutually exclusive forces.

In the synthetic hybrid lineage, maternal (*X. maculatus*) alleles are concentrated within a region of approximately 10 Mb on LG2 after about 100 backcross generations. Such a concentration is not seen elsewhere in the genome, or in the wild-type non-selected hybrid lineage. We suggest that a combination of two proximate mechanisms may have contributed to the retention of this unexpectedly large maternal genomic block. Firstly, a mechanism likely contributing to the generation and or maintenance of this genomic pattern is a translocation event accompanied by recombination suppression. Specifically, potentially sometime during the establishment of the selected backcross line the region of LG21 known to encompass the black pigmentation locus *Sd* including the *xmrk* oncogene, the female sex-determining locus, and the red colour pattern locus *Dr* was translocated into the region on LG2. By imposing selection for these genes (a small region of former LG21^46^), the genes and the surrounding non-recombining regions on LG2 are transmitted as a large block. Second, theoretical predictions suggest that blocks of an introgressing genome are expected to be longer around selected loci^48^. Therefore, due to artificial selection of two genes, sometime during the early backcross generations a large maternally derived block encompassing the selected genes may have been maintained. We note that the higher proportion of maternal SNPs retained elsewhere in the genome may be due to the selection of fertile individuals in each backcross generation, and therefore the purging of incompatible combinations of alleles while an excess of maternal alleles are left.

Given that the core of the retained genomic block originates from the X chromosome and includes the sex-determining locus (from *X. maculatus*), the genomic consequences of this form of hybridisation followed by repeated backcrossing and selection appears to include the formation of a new sex chromosome. Our results strongly suggest that an X chromosome from the platyfish, *X. maculatus*, was introgressed into the genome of the swordtail, *X. hellerii*, which has a ZW sex chromosome system. After approximately 100 generations of backcrossing, the platyfish X-chromosome derived sex-determining region (X-SD) has moved to what was previously an autosomal location. While in the purebred platyfish homozygosity at the *Sd* region of X is required for female development, in the BC_100_ heterozygosity is sufficient. Thus the recombinant neo-sex chromosome behaves like a W with a dominant effect on SD. In the backcrosses, only male *X. hellerii* were used, and the almost exclusive female sex of the fish expressing the selected colour loci on LG2 in the highest backcross generations show that the Z-chromosome (LG21) in *X. hellerii* has no male determining power. Our observation that the sex-determining region of the X was translocated to an autosome provides the first empirical evidence of a heterologous transition. Additionally, at its new chromosomal position the former X-chromosome specific region is located within a large region of retained maternal sequences, indicating that the loss of recombination accompanied the establishment of the novel W chromosome. This formation of a new sex chromosome under a hybridisation with backcrossing scenario highlights the possibility that the variation in genetic sex-determining mechanisms found in the genus *Xiphophorus*, which include simple male and female heterogametic systems together with more complicated situations^24^, may be contributed to by the many hybridisation events inferred among species in this genus. More specifically, this formation of a new sex chromosome provides a key target for future functional studies, and for further investigations of the *Xiphophorus* species thought to have arisen through hybridisation with backcrossing in the wild. For example, could this new sex chromosome play a role in reproductive isolation? And do the sex chromosomes of wild hybrids play similar roles?

The X chromosome has been found to play a special role in reproductive isolation in many species, most likely due to Haldane’s rule, meiotic drive or faster evolution (‘faster X’)^49^. However, to date a definitive role for the X chromosome in reproductive isolation and speciation remains elusive in *Xiphophorus*. In another *Xiphophorus* species, *Xiphophorus* nezahualcoyotl, that exhibits significant hybrid ancestry, it has been indicated that the putative X chromosome has lower levels of coding and non-coding introgression than average, although reportedly not a clear outlier from other chromosomes^27^. Sex chromosome systems in closely related species of fish are generally highly variable, suggesting changes occur rapidly in these systems, and neo-sex chromosomes or novel SD regions have been reported in natural populations^50–55^.

More broadly, further studies of hybridisation in this genus are imperative for fully understanding the genomic and adaptive consequences of the process, including at different time scales. Importantly, recent advances have been made in the development and application of genomic methods for detecting hybridisation and inferring individual ancestries^56–58^. Indeed, fine-scale signals of hybridisation can be detected using genomic analyses that allow the identification of introgressed genes and gene combinations which may be important in adaptation (e.g. refs. ^59–61^). Also, long-read de novo assemblies of various species allow more refined measures of genomic introgression events, a necessary measure to develop evolutionary theories of molecular mechanisms. Broadly, research to date suggests that hybridisation can be a mechanism for co-opting functional elements from another genome^61,62^. For instance, modern humans carry genomic regions derived from Neanderthal ancestors including loci involved in skin and hair phenotypes suggested to be adaptive for humans migrating to non-African environments^61,62^. Importantly, previous studies have suggested that the rate of hybrid genome stabilisation varies across taxa and is dependent on demographic differences, selection or strong genetic drift^57^. In rare and extreme cases, whole genome hybridisations can alter the course of major traits such as meiosis^38^.

In the current study, by using an experimental backcross for over 100 generations we could directly observe the genomic consequences of hybridisation with backcrossing. Importantly this was done with two parental species with different sex (determining) chromosome systems, and where we strongly selected for distinct phenotypes. It is likely that a combination of different processes drove the observed genomic outcomes, including exogenous selection (i.e. artificial selection for the pigmentation phenotype and female sex), and endogenous selection such as gene–gene interactions. Further, the genomic patterns observed here could have come about through a number of different scenarios that require future investigation. For instance, the patterns may reflect the potential for a connection between chromosomal organisation and adaptive evolution in this system. Alternatively, and potentially equally parsimoniously, the translocation event may have also been neutral. Recombination suppression associated with chromosomal rearrangement can promote local adaptation and the accumulation of genetic incompatibilities between species (reviewed in refs. ^63,64^). In *Helianthus* sunflowers, where large linkage blocks appearing to resist recombination were observed in both experimental and ancient hybrids, gene interactions were invoked as the most likely explanation for the observed concordance in genomic pattern^65^. In another more recent study where introgression and local adaptation in two poplar species were investigated using fine-scale genomic techniques, admixed individuals of one species harbour a telomeric region on one chromosome which had introgressed from the other species, and this region was found to contain several candidate genes for local adaptation^59^. Interestingly, a paralogous block of genes on another chromosome showed no signs of introgression or signatures of selection^59^. Natural hybridisation between several species in the genus *Xiphophorus* has been inferred and has reportedly occurred across different timescales, including contemporary and more ancient events^27,34,36,37,39^, suggesting hybridisation, such as the scenario investigated in our study, may be a key contributor to the evolutionary history of this group of fishes.

## Methods

### Sex determination in *Xiphophorus hellerii*

A total of 30 female and 30 male *X. hellerii* individuals from the same strain (origin Rio Lancetilla), which were also used for the backcrossing experiment, were genotyped to identify sex-related markers, and thus characterize the sex determination system of this swordtail species strain.

### Sequencing and data filtering

Genomic DNA was extracted from fin clips using standard phenol–chloroform extraction^66^, followed by RNase treatment. DNA quality of each sample was assessed by agarose gel electrophoresis and quantified using a Qubit v2.0 fluorometer (Life Technologies, Darmstadt, Germany). Approximately 100 ng of DNA template of each sample was used to construct a single quaddRAD^67^ library (PstI and MspI were used as rare and frequent restriction enzymes, respectively), size selected from 450 to 550 bp using a Pippin Prep platform (Sage Sciences, Beverly, USA) and paired-end sequenced in an Illumina HiSeq2500 (rapid run mode, 2 × 151 cycles).

The raw fastq files obtained from one Illumina lane (NCBI’s SRA database, accession no. PRJNA493969) were first processed using the *clone_filter* module implemented in the Stacks v1.46^68,69^ package to identify and remove duplicate reads. The retained sequence data, stripped of the four random bases at the 5′ end of each paired read, was then separated by the dual index inner barcodes using the Stacks’ *process_radtags* script (option: *–inline_inline*) with no quality filters applied. Next, the sequences were processed by the dDocent v2.2.25 pipeline^70^, with default parameters, in order to get the individuals’ genotypes. For this RAD data set, the dDocent pipeline was preferred to Stacks because of its superior ability to deal with paired-end reads when calling genotypes. Briefly, dDocent calls Trimmomatic v0.36^71^, bwa v0.7.15^72^ and Freebayes v1.1.0^73^ to respectively quality control the raw reads, align the filtered sequences to the reference genome and infer individuals’ genotypes at polymorphic loci. Trimmomatic was used to remove bases with a quality score <20 from the beginning and end of reads, and additionally trim bases with an average quality score <10 in a sliding 5 bp window. The ‘mem’ algorithm implemented in bwa was applied setting the match score value (-A) to 1, the penalty for a mismatch (-B) to 4 and the gap penalty (-O) to 6. Freebayes was used to call genotypes setting a minimum mapping and base quality score to 10.

### Sequence variation analysis: filtering and GWAS of sex in *X. hellerii*

The raw VCF variant file was obtained by setting the *X. maculatus* v4.4.2 as the reference genome^46^. In this version of the *X. maculatus* genome, the original scaffolds^43^ were tiled along a dense genetic map to create chromosome-length genome assemblies. The VCF file was filtered using VCFtools v0.1.15^74^ to retain loci present in a minimum of 80% of the individuals and having mapping quality (MQ) > 30. Further stringent filtering was applied using the *dDocent_filters* script (http://ddocent.com/filtering/) that relies on both VCFtools and the *vcffilter* module in the vcflib package (https://github.com/vcflib/). A total of 65,417 SNPs passed the quality control applied and were used for the GWAS and the F_ST_ analyses (the full set of filters, and the number of sites removed at each step, are reported in Supplementary Table 3; the individuals’ genotypes were deposited into the Dryad Data Repository: 10.5061/dryad.7h54h66).

The final VCF file was used to perform a standard genome-wide association analysis using PLINK v1.90b4.9^75^ setting sex as the binary case/control variable. The −log_10_ *p*-value (obtained with a Fisher’s exact test) of each SNP was shown as a function of genomic position on the 24 *X. maculatus* LGs^46^ in a Manhattan plot constructed with the R package qqman v0.1.4^76^. The threshold for genome-wide significance was set at a *p*-value = 7.64e^−7^ after Bonferroni correction for multiple comparisons (0.05/65,417 variants). To identify highly divergent, potential candidate sex-chromosome regions using an additional line of investigation, the program VCFtools was used to calculate F_ST_-values between males and females across different non-overlapping sliding windows (1, 10 and 100 Kb). As a suggestive threshold for significance, we used the upper 1% percentile distribution of the per window F_ST_ values (1 Kb window: F_ST_ = 0.184; 1 Kb window: F_ST_ = 0.181; 1 Kb window: F_ST_ = 0.157).

### Coverage analysis to identify sex association in *X. hellerii*

As an alternative and independent approach to identify and verify potential sex-associated SNPs, we analysed mapped sequence coverage information from *X. hellerii* males and females to identify loci located in the non-recombining and recombination restricted regions of the sex chromosomes. We first created a set of reference loci using the *ustacks* module of the Stacks package in default mode, but setting the minimum depth of coverage required to create a stack (-m) to 5. As a reference for aligning the RAD loci, we used the *X. hellerii* genome v3.0.1 (NCBI accession number: GCA_001443345.1)^47^ that is derived from a single female, strain Sarabia, from the Xiphophorus Genetic Stock Center (TX, USA). Existing evidence suggested that the female is the heterogametic sex in this species; therefore, because the genome of *X. hellerii* was generated using a single female individual, using the *X. hellerii* genome would allow us to recover a higher number of W-linked loci than using the *X. maculatus* genome. We used this approach to confirm the findings of the statistically more robust GWAS approach, and possibly to identify specific *X. hellerii* sex-determining regions (e.g. autosomal modifiers). The total 70,763 loci, extracted from the Stacks’ catalogue formatted in fasta format (deposited into the Dryad Data Repository: 10.5061/dryad.7h54h66), were used as a reference for the alignment of the sequence set of each sample carried out with Bowtie2 v2.3.0^77^ in ‘--very-sensitive’ mode. To avoid redundant information, only one of the paired loci was retained to serve as a reference. Next, raw coverage values—i.e. the number of reads mapping to each locus—were extracted from each individual mapping file using SAMtools v1.2.1^78^ *idxstats* and normalized using the Median Ratio Normalization (MRN) function implemented in DESeq2 v1.8.1^79^.

We then calculated the mean coverage for each locus (including males and females), but first removed loci with coverage below 3 and above 400 because they most likely indicate mapping errors and repetitive elements, respectively (the number of loci was reduced to 29,444 after the coverage filters were applied). We assumed a ZW sex-determination system is operating in this system, given the results from our sequence variation analysis (see the previous paragraph), and from the filtered coverage data set we selected (1) potentially W-linked female-specific loci and (2) Z-linked loci. According to expectations, W-linked loci should be present only in females, the heterogametic sex, so we applied coverage filters to satisfy this condition (mean coverage in females >7; mean coverage in males <2). On the other hand, the Z-linked loci are expected to be present in both sexes, but they should have twice the coverage in males than in females, as they have two copies of the Z chromosome (coverage filter to identify these loci: mean coverage ratio in male/female >1.9). We note that this type of filtering criteria is dependent on the sequencing depth of coverage used in the experiment, and the use of these arbitrary filters means the results should be interpreted with caution. However, the main purpose of the coverage analysis was to confirm the results of the GWAS analysis. Finally, we used a two-tailed Fisher’s exact test to test for over-representation of these candidate sex-chromosome loci in the *X. maculatus* LGs that were associated with sex (SNP analysis).

### Synthetic hybrids: laboratory cross experiment

Laboratory crosses were conducted which mimic the hybridisation with backcrossing evolutionary scenario that has potentially given rise to at least two *Xiphophorus* fish species, and may have contributed to the ancestry of other taxa. We sampled both first generation and approximately 100th generation backcrossed individuals that had been artificially selected for two pigmentation phenotypes. We note that all sampled backcross generations were raised under the same standard laboratory conditions, therefore excluding any environmental effects.

In these laboratory crosses, one parental species is *X. maculatus*, where the strain Jp163A (origin Rio Jamapa) was used, which is also the same strain from which the reference genome was produced^43^. This strain has been kept as a brother–sister mating line for more than 100 generations. The other parental species used in the laboratory crosses is *X. hellerii*. The stock is derived from fish collected from the Rio Lancetilla and has been kept for over 50 years in closed colony breeding. The *X. hellerii* stock has been through several bottlenecks (due to the breakdown of the stock) where only a handful of fish or even a single pair was used to regenerate the stock. Both parental species are representatives of the two clades that were earlier shown to have been involved in the initial hybridisation event generating *X. clemenciae* and *X. monticolus*^33,36,37^.

The first generation hybrid cross was between a female *X. maculatus* and a male *X. hellerii*. Next, a backcross was made between an F_1_ female, carrying the pigmentation phenotypes intended for selection and an *X. hellerii* male. Females were then selected for each successive backcross according to the two colour markers. One is the erythrophore pigmentation pattern *dorsal red*, *Dr*, which causes a dark red colouration of the dorsal fin and in hybrids extends from there over the whole body and the unpaired fins. The second pattern is caused by the closely linked macromelanophore pattern locus *spotted dorsal*, *Sd*. It is expressed as jet-black pigmentation spots at the base of the dorsal fin in purebred *X. maculatus*. In the genetic background of *X. hellerii*, the macromelanophores cover the whole dorsal fin, and from there invade the dorsal and caudal body compartment of the fish. As an effect of the *Sd* locus component, *xmrk* oncogene^74^, the macromelanophore pattern in the genetic background of *X. hellerii* is enhanced to melanotic hyperpigmentation that can progress to malignant melanoma (for an overview of the pigmentation genetics of platyfish/swordtail hybrids, see ref. ^40^). *Dr* and *Sd/xmrk* are closely linked on the X-chromosome of the platyfish corresponding to linkage group 21^43,46^. In the serial backcross experiment, each generation consists of 50% fish with and 50% without the chromosome region where both genes are located, designated as ‘pigm’ or ‘wt’ group. Selecting fish with the two linked colour markers for breeding mimics natural selection for a certain phenotype. Therefore, we expect fish with the pigmentation phenotype to carry the parental platyfish genetic region of the X-chromosome. Backcrossing was continued in this fashion for at least 100 generations (more than 30 years of laboratory crosses have been performed, counting was terminated after backcross 50 and on average three backcross generations were produced per year). For the approximate 100th generation backcross offspring, we expect fish carrying the pigmentation genes (further on referred to as BC_100__pigm) to be enriched for genetic markers of platyfish LG21. By contrast, segregants not carrying the colour genes (wild type pigmented, further on referred to as BC_100__wt) should not be enriched for platyfish sequences of this region of the genome.

### Sequencing, filtering and genotype calling

Genomic DNA was extracted from pooled organs (brain, eyes, gills, liver, spleen, kidney) of the two parental species (two males and two female individuals each for *X. maculatus* and *X. hellerii*), four F_1_ females, 16 first generation backcross individuals and 18 individuals generated after about 100 backcross generations. DNA extraction was carried out using the standard phenol–chloroform extraction^66^. The DNA quality of each sample was determined by agarose gel electrophoresis and quantified using a Qubit v2.0 fluorometer. Approximately 300 ng of DNA template of each sample was used to construct double-digest restriction site-associated DNA (ddRAD)^80^ libraries following the modifications introduced in ref. ^81^. DNA digestion was carried out using the restriction enzymes PstI (rare cutter) and MspI (frequent cutter).

A single ddRAD library containing 46 barcoded individuals (see Supplementary Data 3 for details) was constructed, size selected from 360 to 430 bp using a Pippin Prep system and single-end sequenced in an Illumina HiSeq2500 using rapid run mode with 151 cycles (raw reads were deposited in the NCBI’s SRA database, accession no. PRJNA493969).

The *process_radtags* script implemented in the Stacks package was used for individual demultiplexing and for filtering erroneous and low-quality reads (options: -c –q). After this process, an average of 2.95 million (*X. maculatus*) and 2.08 million (*X. hellerii*) sequences were obtained for the parents; an average of 3.05, 1.91 and 2.34 million sequences were obtained for the F_1_, BC_1_ and BC_100_ individuals, respectively (see Supplementary Data 3). The sequence length of each read was 146 bp after removing its 5-bp barcode.

Filtered reads were individually aligned to the anchored version of the *X. maculatus* genome^46^ using Bowtie2 with default end-to-end mode. SAMtools was then used to filter the mapping result by retaining only sites with high quality score (MQ ≥ 30). Loci construction and genotype calling were performed using the Stacks’ *ref_map.pl* pipeline requiring a minimum of five reads (- m 5) to form a locus and calling genotypes at a 5% significance level using the bounded error model (upper bound of 0.05). The allelic variants (SNPs) were exported in VCF format using the Stacks’ *populations* module.

### Sorting of parental alleles in the laboratory cross dataset

An in-house Perl script was used to identify the subset of sites fixed for alternative alleles in the two parental species (*X. maculatus and X. hellerii*) of the laboratory cross following these criteria: (1) the individuals’ genotypes are homozygous within each parental species, but they have different allelic variants; (2) the sites selected previously are heterozygous including both parentals’ allelic variants in F_1_ individuals; (3) at these sites, all BC_1_ and BC_100_ individuals have at least one paternal (*X. hellerii*) allele. After these filtering steps, we obtained a total of 34,632 SNPs (the individuals’ genotypes were deposited into the Dryad Data Repository: 10.5061/dryad.7h54h66).

### Tests for excess ancestry

As we focused on SNPs fixed between the parental species (see above), each SNP was fully informative about ancestry. Thus, we were able to directly calculate the frequency of platyfish ancestry in the BC_100_ lines. We then used a stochastic simulation to develop null expectations for the frequency of platyfish ancestry expected in the absence of selection after 100 generations of backcrossing. We sampled platyfish ancestry frequencies each generation stochastically as *y*_t_ = binomial(*p*_t−1_ * 0.5, 2*N*) and then set *p*_t_ = *y*_t_/2*N*. Here, *y*_t_ is the number of platyfish alleles in the sample of *N* diploid hybrid fish and *p*_t_ and *p*_t−1_ are the ancestry frequencies in the current and previous generation, respectively. The expectation for the binomial is reduced by half each generation as the BC line is backcrossed to *X. hellerii*. Using this iterative process and starting from *p*_0_ = 0.5 (F_1_s), we simulated expectations for 100 generations of backcrossing with *N* = 10 or 15 hybrid fish per generation (conservative estimates of the average number of fish used to maintain the line over time). We repeated this procedure 10,000 times. In all cases, we found expected platyfish ancestry at generation 100 of *p*_100_ = 0.

### Ethical statement

All animals were kept and sampled in accordance with the applicable EU and national German legislation governing animal experimentation. In particular, all experimental protocols were approved through an authorization (568/300-1870/13) of the Veterinary Office of the District Government of Lower Franconia, Germany, in accordance with the German Animal Protection Law (TierSchG).

## Electronic supplementary material

Supplementary Information

Description of Additional Supplementary Files

Supplementary Data 1

Supplementary Data 2

Supplementary Data 3

Supplementary Data 4

Supplementary Data 5

## Data Availability

Raw Illumina sequences were deposited in the NCBI’s Sequence Read Archive (SRA) database with accession no. PRJNA493969. The genotypes of each data set that we described in the manuscript (VCF format) and the Stacks' loci we used in the coverage analysis (FASTA format) have been uploaded to the Dryad Digital Repository (10.5061/dryad.7h54h66). A reporting summary for this article is available as a Supplementary Information file.

## References

[1] Bull, J. J. *Evolution of Sex Determining Mechanisms* (Benjamin Cummings, Menlo Park, CA, USA, 1983).

[2] Phillips RB, Konkol NR, Reed KM, Stein JD (2001). Chromosome painting supports lack of homology among sex chromosomes in *Oncorhynchus*, *Salmo*, and *Salvelinus* (Salmonidae). Genetica.

[3] Woram RA (2003). Comparative genome analysis of the primary sex-determining locus in salmonid fishes. Genome Res..

[4] Mank JE, Promislow DE, Avise JC (2006). Evolution of alternative sex-determining mechanisms in teleost fishes. Biol. J. Linn. Soc..

[5] Volff JN, Nanda I, Schmid M, Schartl M (2007). Governing sex determination in fish: regulatory putsches and ephemeral dictators. Sex. Dev..

[6] Mank JE, Avise JC (2009). Evolutionary diversity and turn-over of sex determination in teleost fishes. Sex. Dev..

[7] Kitano J, Peichel CL (2012). Turnover of sex chromosomes and speciation in fishes. Environ. Biol. Fish..

[8] Miura I (2008). An evolutionary witness: the frog *Rana rugosa* underwent change of heterogametic sex from XY male to ZW female. Sex. Dev..

[9] Stöck M (2011). A cryptic heterogametic transition revealed by sex-linked DNA markers in Palearctic green toads. J. Evol. Biol..

[10] Gamble T (2015). Restriction site-associated DNA sequencing (RAD-seq) reveals an extraordinary number of transitions among gecko sex-determining systems. Mol. Biol. Evol..

[11] Graves JAM (2008). Weird animal genomes and the evolution of vertebrate sex and sex chromosomes. Annu. Rev. Genet..

[12] Schartl M (2004). Sex chromosome evolution in non-mammalian vertebrates. Curr. Opin. Genet. Dev..

[13] Blaser O, Neuenschwander S, Perrin N (2014). Sex-chromosome turnovers: the hot-potato model. Am. Nat..

[14] van Doorn GS, Kirkpatrick M (2010). Transitions between male and female heterogamety caused by sex-antagonistic selection. Genetics.

[15] Bachtrog D (2014). Sex determination: why so many ways of doing it?. PLoS Biol..

[16] Herpin A, Schartl M (2015). Plasticity of gene-regulatory networks controlling sex determination: of masters, slaves, usual suspects, newcomers, and usurpators. EMBO Rep..

[17] Schartl M (2015). Sex determination by multiple sex chromosomes in *Xenopus tropicalis*. Proc. Natl. Acad. Sci. U.S.A..

[18] Haldane JBS (1922). Sex ratio and unisexual sterility in animal hybrids. J. Genet..

[19] Mallet J (2005). Hybridization as an invasion of the genome. Trends Ecol. Evol..

[20] Nanda I, Hornung U, Kondo M, Schmid M, Schartl M (2003). Common spontaneous sex-reversed XX males of the medaka *Oryzias latipes*. Genetics.

[21] Wilson CA (2014). Wild sex in zebrafish: loss of the natural sex determinant in domesticated strains. Genetics.

[22] Woolcock B (2006). Allele-specific marker generation and linkage mapping on the *Xiphophorus* sex chromosomes. Zebrafish.

[23] Schultheis C, Böhne A, Schartl M, Volff JN, Galiana-Arnoux D (2009). Sex determination diversity and sex chromosome evolution in Poeciliid fish. Sex. Dev..

[24] Schartl, M., Galiana-Arnoux, D., Schultheis, C., Böhne, A. & Volff, J.-N. *Ecology and Evolution of Poecliid Fishes* (eds Evans, J. P. et al.) (University of Chicago Press, Chicago, IL, USA, 2011).

[25] Kallman, K. D. *Evolutionary Genetics of Fishes* (ed Turner, B. J.) 95–171 (Springer, New York, NY, USA, 1984).

[26] Volff JN, Schartl M (2001). Variability of genetic sex determination in poeciliid fishes. Genetica.

[27] Schumer M, Cui R, Powell DL, Rosenthal GG, Andolfatto P (2016). Ancient hybridization and genomic stabilization in a swordtail fish. Mol. Ecol..

[28] Gordon M (1937). Heritable color variations in the Mexican swordtail-fish aquarium species as the *Drosophila* of fish genetics. J. Hered..

[29] Atz, J. W. Effects of hybridization on pigmentation in fishes of the genus *Xiphophorus*. *Zoologica* **47**, 153–181 (1962).

[30] Häussler G (1928). Über Melanombildungen bei Bastarden von *Xiphophorus helleri* und *Platypoecilus maculatus* var. Rubra. J. Mol. Med..

[31] Gordon M (1927). The genetics of a viviparous top-minnow Platypoecilus; the inheritance of two kinds of melanophores. Genetics.

[32] Koßwig C. (1927). Über Bastarde der Teleostier Platypoecilus und Xiphophorus. Zeitschrift für Induktive Abstammungs- und Vererbungslehre.

[33] Meyer A, Salzburger W, Schartl M (2006). Hybrid origin of a swordtail species (Teleostei: *Xiphophorus clemenciae*) driven by sexual selection. Mol. Ecol..

[34] Culumber ZW (2011). Replicated hybrid zones of *Xiphophorus* swordtails along an elevational gradient. Mol. Ecol..

[35] Jones JC, Perez-Sato JA, Meyer A (2012). A phylogeographic investigation of the hybrid origin of a species of swordtail fish from Mexico. Mol. Ecol..

[36] Jones JC, Fan S, Franchini P, Schartl M, Meyer A (2013). The evolutionary history of *Xiphophorus* fish and their sexually selected sword: a genome-wide approach using restriction site-associated DNA sequencing. Mol. Ecol..

[37] Kang JH, Schartl M, Meyer A (2013). Comprehensive phylogenetic analysis of all species of swordtails and platies (Pisces: Genus *Xiphophorus*) uncovers a hybrid origin of a swordtail fish, *Xiphophorus monticolus*, and demonstrates that the sexually selected sword originated in the ancestral lineage of the genus, but was lost again secondarily. BMC Evol. Biol..

[38] Schumer M (2018). Natural selection interacts with recombination to shape the evolution of hybrid genomes. Science.

[39] Cui R (2013). Phylogenomics reveals extensive reticulate evolution in *Xiphophorus* fishes. Evolution.

[40] Schartl M (2008). Evolution of Xmrk: an oncogene, but also a speciation gene?. Bioessays.

[41] Rosenthal GG, Ryan MJ (2011). Conflicting preferences within females: sexual selection versus species recognition. Biol. Lett..

[42] Meyer A, Morrissey JM, Schartl M (1994). Recurrent origin of a sexually selected trait in *Xiphophorus* fishes inferred from a molecular phylogeny. Nature.

[43] Schartl M (2013). The genome of the platyfish, *Xiphophorus maculatus*, provides insights into evolutionary adaptation and several complex traits. Nat. Genet..

[44] Peters VG (1964). Vergleichende Untersuchungen an drei Subspecies von *Xiphophorus helleri* Heckel (Pisces): (Morphologische Merkmale, geschlechtliche Differenzierung, Wachstum und Geschlechtsverhältnisse). J. Zool. Syst. Evolut. Res..

[45] Kazianis, S. *Viviparous Fishes* (eds Uribe, M. C. & Grier, H.) 381–400 (New Life Publications, Mexico, 2005).

[46] Amores A (2014). A RAD-tag genetic map for the platyfish (*Xiphophorus maculatus*) reveals mechanisms of karyotype evolution among teleost fish. Genetics.

[47] Shen Y (2016). *X. couchianus* and *X. hellerii* genome models provide genomic variation insight among *Xiphophorus* species. BMC Genomics.

[48] Sedghifar A, Brandvain Y, Ralph P (2016). Beyond clines: lineages and haplotype blocks in hybrid zones. Mol. Ecol..

[49] Presgraves DC (2008). Sex chromosomes and speciation in *Drosophila*. Trends Genet..

[50] Kitano J (2009). A role for a neo-sex chromosome in stickleback speciation. Nature.

[51] Ross JA, Urton JR, Boland J, Shapiro MD, Peichel CL (2009). Turnover of sex chromosomes in the stickleback fishes (Gasterosteidae). PLoS Genet..

[52] Kondo M (2006). Genomic organization of the sex-determining and adjacent regions of the sex chromosomes of medaka. Genome Res..

[53] Tanaka K, Takehana Y, Naruse K, Hamaguchi S, Sakaizumi M (2007). Evidence for different origins of sex chromosomes in closely related oryzias fishes: substitution of the master sex-determining gene. Genetics.

[54] Roberts RB, Ser JR, Kocher TD (2009). Sexual conflict resolved by invasion of a novel sex determiner in Lake Malawi cichlid fishes. Science.

[55] Ser JR, Roberts RB, Kocher TD (2010). Multiple interacting loci control sex determination in Lake Malawi cichlid fish. Evolution.

[56] Payseur BA, Rieseberg LH (2016). A genomic perspective on hybridization and speciation. Mol. Ecol..

[57] Abbott RJ, Barton NH, Good JM (2016). Genomics of hybridization and its evolutionary consequences. Mol. Ecol..

[58] Gompert Zachariah, Mandeville Elizabeth G., Buerkle C. Alex (2017). Analysis of Population Genomic Data from Hybrid Zones. Annual Review of Ecology, Evolution, and Systematics.

[59] Suarez-Gonzalez A (2016). Genomic and functional approaches reveal a case of adaptive introgression from *Populus balsamifera* (balsam poplar) in *P. trichocarpa* (black cottonwood). Mol. Ecol..

[60] VonHoldt BM, Kays R, Pollinger JP, Wayne RK (2016). Admixture mapping identifies introgressed genomic regions in North American canids. Mol. Ecol..

[61] Sankararaman S (2014). The genomic landscape of Neanderthal ancestry in present-day humans. Nature.

[62] Vernot B, Akey JM (2014). Resurrecting surviving neandertal lineages from modern human genomes. Science.

[63] Faria R, Navarro A (2010). Chromosomal speciation revisited: rearranging theory with pieces of evidence. Trends Ecol. Evol..

[64] Ortiz-Barrientos D, Engelstädter J, Rieseberg LH (2016). Recombination rate evolution and the origin of species. Trends Ecol. Evol..

[65] Rieseberg L, Sinervo B, Linder C, Ungerer M, Arias D (1996). Role of gene interactions in hybrid speciation: evidence from ancient and experimental hybrids. Science.

[66] Blin N, Stafford DW (1976). A general method for isolation of high molecular weight DNA from eukaryotes. Nucleic Acids Res..

[67] Franchini P, Monné Parera D, Kautt AF, Meyer A (2017). quaddRAD: a new high-multiplexing and PCR duplicate removal ddRAD protocol produces novel evolutionary insights in a nonradiating cichlid lineage. Mol. Ecol..

[68] Catchen J, Hohenlohe PA, Bassham S, Amores A, Cresko WA (2013). Stacks: an analysis tool set for population genomics. Mol. Ecol..

[69] Catchen JM, Amores A, Hohenlohe P, Cresko W, Postlethwait JH (2011). Stacks: building and genotyping loci de novo from short-read sequences. G3.

[70] Puritz JB, Hollenbeck CM, Gold JR (2014). dDocent: a RADseq, variant-calling pipeline designed for population genomics of non-model organisms. PeerJ.

[71] Bolger Anthony M., Lohse Marc, Usadel Bjoern (2014). Trimmomatic: a flexible trimmer for Illumina sequence data. Bioinformatics.

[72] Li H., Durbin R. (2009). Fast and accurate short read alignment with Burrows-Wheeler transform. Bioinformatics.

[73] Garrison, E. & Marth, G. Haplotype-based variant detection from short-read sequencing. Preprint at arXiv:1207.3907v2 [q-bio.GN] (2012).

[74] Danecek P (2011). The variant call format and VCFtools. Bioinformatics.

[75] Purcell S (2007). PLINK: A tool set for whole-genome association and population-based linkage analyses. Am. J. Hum. Genet..

[76] Turner, S. D. qqman: an R package for visualizing GWAS results using Q–Q and Manhattan plots. *bioRxiv* 1–2. 10.1101/005165 (2014).

[77] Langmead B, Salzberg SL (2012). Fast gapped-read alignment with Bowtie 2. Nat. Methods.

[78] Li H (2009). The sequence alignment/map format and SAMtools. Bioinformatics.

[79] Love, M. I., Huber, W. & Anders, S. Moderated estimation of fold change and dispersion for RNA-seq data with DESeq2. *Genome Biol*. **15**, 550 (2014). 10.1186/s13059-014-0550-8 PMC4302049 25516281

[80] Peterson BK, Weber JN, Kay EH, Fisher HS, Hoekstra HE (2012). Double digest RADseq: an inexpensive method for de novo SNP discovery and genotyping in model and non-model species. PLoS One.

[81] Franchini P (2014). Genomic architecture of ecologically divergent body shape in a pair of sympatric crater lake cichlid fishes. Mol. Ecol..

