# Supplementary material for: Long-term experimental hybridisation results in the evolution of a new sex chromosome in swordtail fish
Source: Nat Commun. 2018 Dec 3;9:5136. doi: 10.1038/s41467-018-07648-2 (PMC6277394; doi:10.1038/s41467-018-07648-2)
Supplement: Supplementary file 1 — Supplementary Information [file 41467_2018_7648_MOESM1_ESM.pdf]

Supplementary Information

Long-term experimental hybridisation results in the evolution of a new sex chromosome in swordtail fish

Franchini et al.

Table of contents

Supplementary Table 1 1

Supplementary Table 2 2

Supplementary Table 3 2

Supplementary Figure 1 3

Supplementary Figure 2 4

Supplementary Figure 3 5

Supplementary Figure 4 6

Supplementary Table 1. Genotypic and phenotypic sex of F_1 hybrids between *X. hellerii* and *X. maculatus.*

X. hellerii* x *X. maculatus			
**Pedigree #**	**Genotype***	**Phenotype**	**Fertility**
483	XZ	male	fertile
730	XZ	male	fertile
	XW	female	sterile
1995	XW	female	sterile
	XZ	male	fertile
	ZY	male	fertile
1997	XW	female	sterile
	XZ	male	fertile
4404	XW	female**	sterile
	XW	female	fertile
	WY	female	fertile
	ZY	male	fertile

X. maculatus* x *X. hellerii			
**Pedigree #**	**Genotype***	**Phenotype**	**Fertility**
6457	XZ	female	fertile
	XZ	male	fertile
	XZ	male	fertile

***determined by pigmentation marker genes.**

****a total of six such fish were obtained in this cross.**

Supplementary Table 2. Sex ratio distribution in the BC_{100} fishes.

Sex	BC_{100}_wt	BC_{100}_pigm
Male	235	10
Female	22	625

Supplementary Table 3. Filtering criteria applied to the quaddRAD dataset with the number of SNPs retained after each step.

Program used to filter loci	Type of filter applied with options used	Number of loci before filtering	Number of loci after filtering
VCFtools	Maximum number of missing genotypes = 20% (--max-missing 0.8)	874,419	317,705
VCFtools	Minor allele count = 3 (--mac 3)	317,705	98,951
VCFtools	Minimum mapping quality (MQ) = 30 (--minQ 30)	98,951	90,452
VCFfilter	Allele balance (AB) at heterozygous loci (AB > 0.25 & AB < 0.75 | AB < 0.01)	90,452	82,223
VCFfilter	Overlapping forward and reverse reads (SAF / SAR > 100 & SRF / SRR > 100 | SAR / SAF > 100 & SRR / SRF > 100)	82,223	71,379
VCFfilter	Read paired status (PAIRED > 0.05 & PAIREDR > 0.05 & PAIREDR / PAIRED < 1.75 & PAIREDR / PAIRED > 0.25 | PAIRED < 0.05 & PAIREDR < 0.05)	71,379	69,410
VCFtools	Quality scores > 2 times the depth (--site-depth --exclude-positions loci_not_satysfying_criteria.txt)	69,410	68,349
VCFtools	Maximum mean depth cutoff = 100 (--max-meanDP 100)	68,349	65,417

Total number of loci after all the filtering applied: 65,417

Supplementary Fig. 1

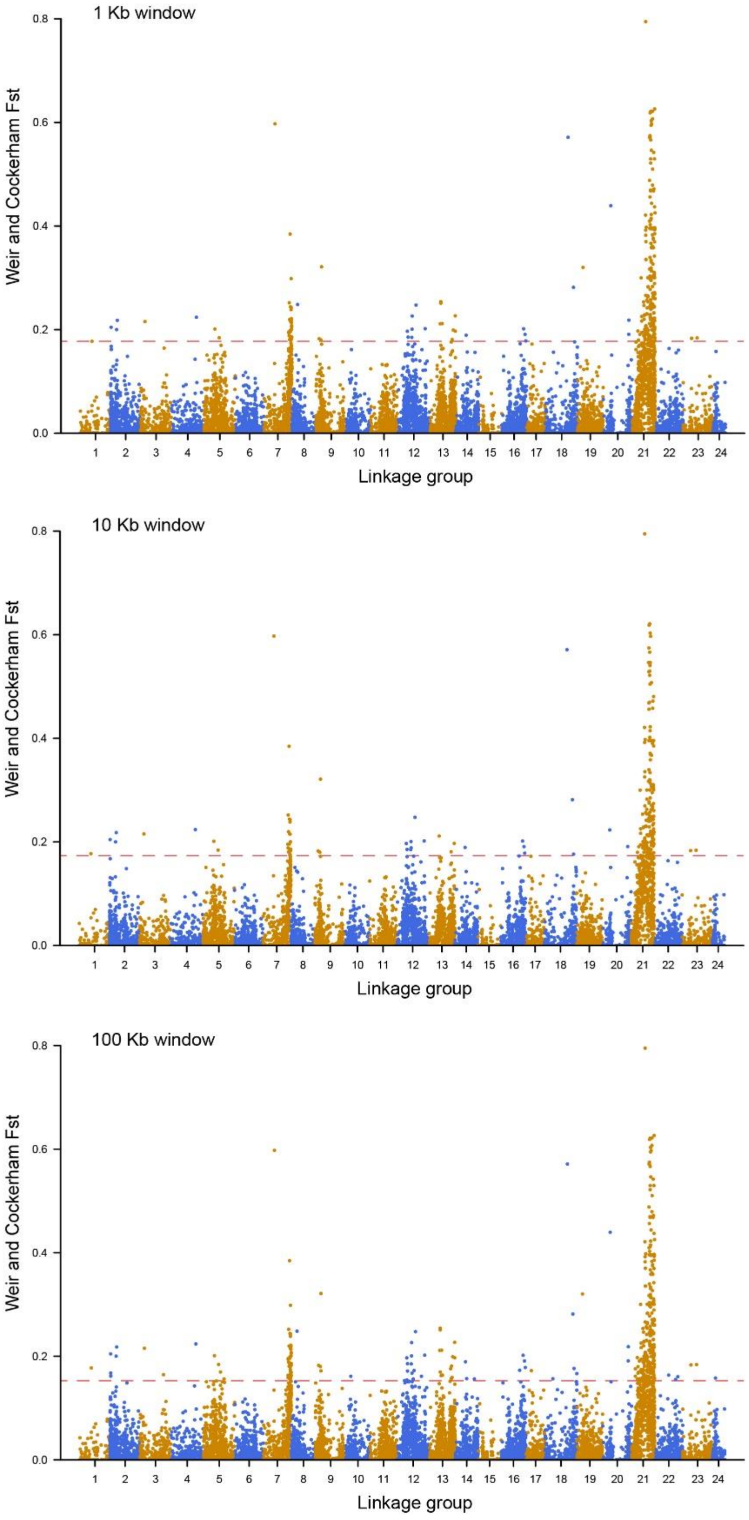

Supplementary Fig. 1. Genome-wide plot of F_{ST}-values calculated at three different sliding windows: 1 Kb, 10 Kb and 100 Kb. F_{ST}-values for loci in odd-numbered LGs are represented by orange dots while blue dots are used for values in even-numbered LGs. The dashed red line represent the upper 1% percentile of the distribution of the per window F_{ST} values (1 Kb window: F_{ST} = 0.184; 10 Kb window: F_{ST} = 0.181; 100 Kb window: F_{ST} = 0.157).

Supplementary Fig. 2

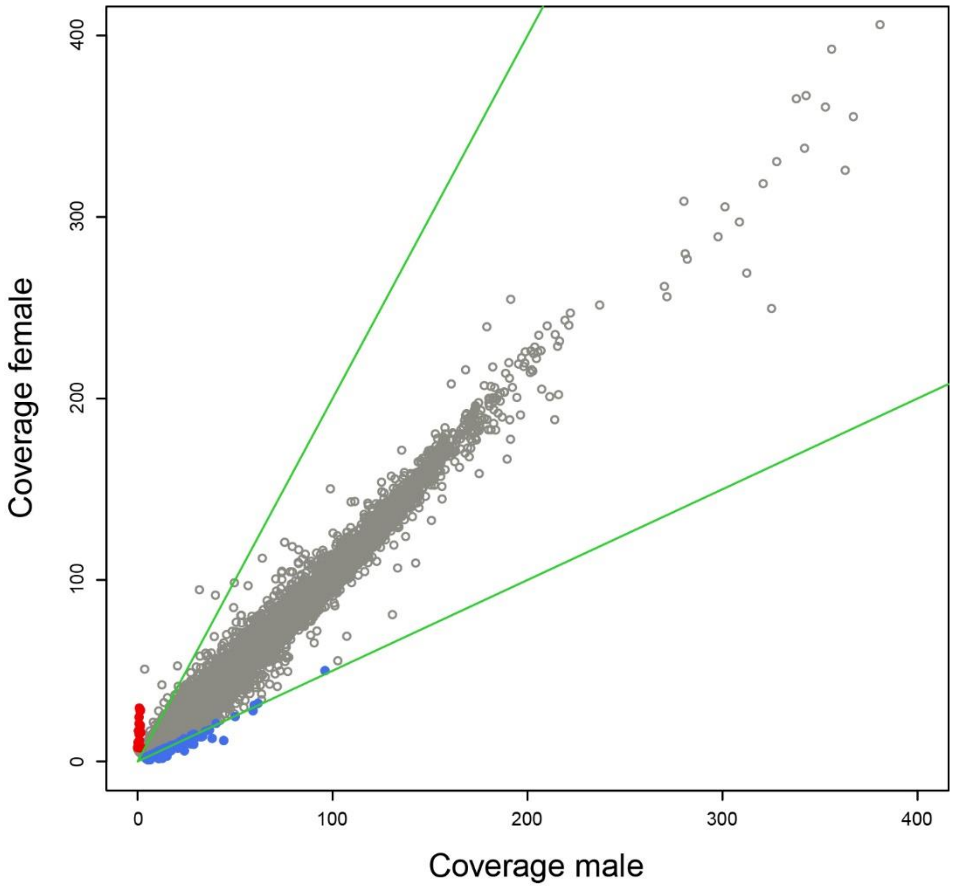

Supplementary Fig. 2. Sequence coverage of the filtered set of RAD loci in female and male *X. hellerii* individuals with no cut off applied. Each dot in the scatter plot represents the average coverage across all males (x-axis) against the average coverage across all females (y-axis) for each of the selected RAD loci. Red dots indicate potential W-linked loci (female-specific loci), while blue dots indicate loci potentially linked to the Z chromosome (twice the coverage in males than in females). The upper and lower green lines show the expected coverage of X-linked and Z-linked loci in a XY and ZW sex determination system, respectively.

Supplementary Fig. 3

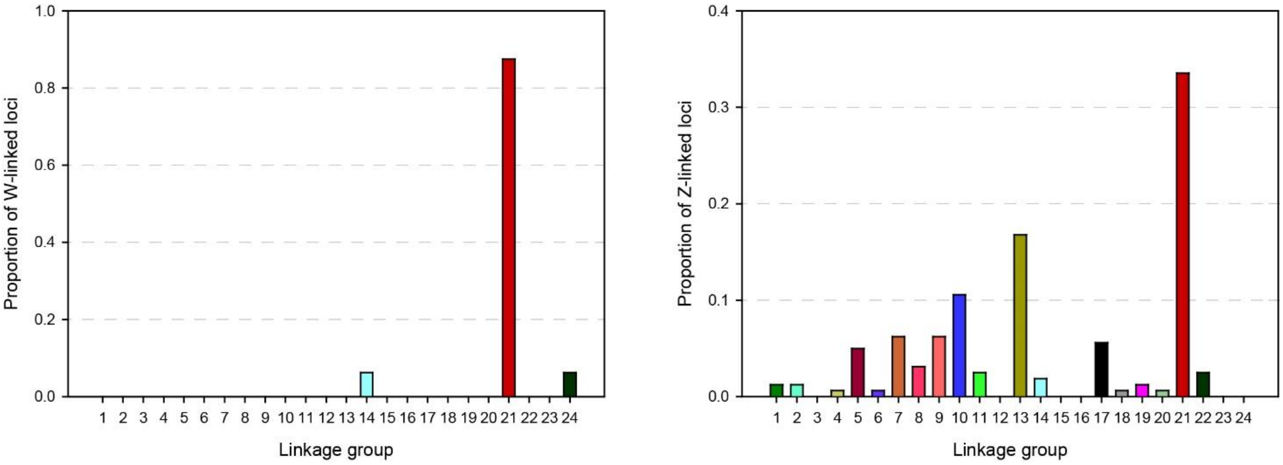

Supplementary Fig. 3. A total of 18 W-linked and 178 Z-linked loci were identified in the coverage analysis of *X. hellerii*. The box plots show the proportion of W-linked (left) and Z-linked (right) loci for each linkage group.

Supplementary Fig. 4

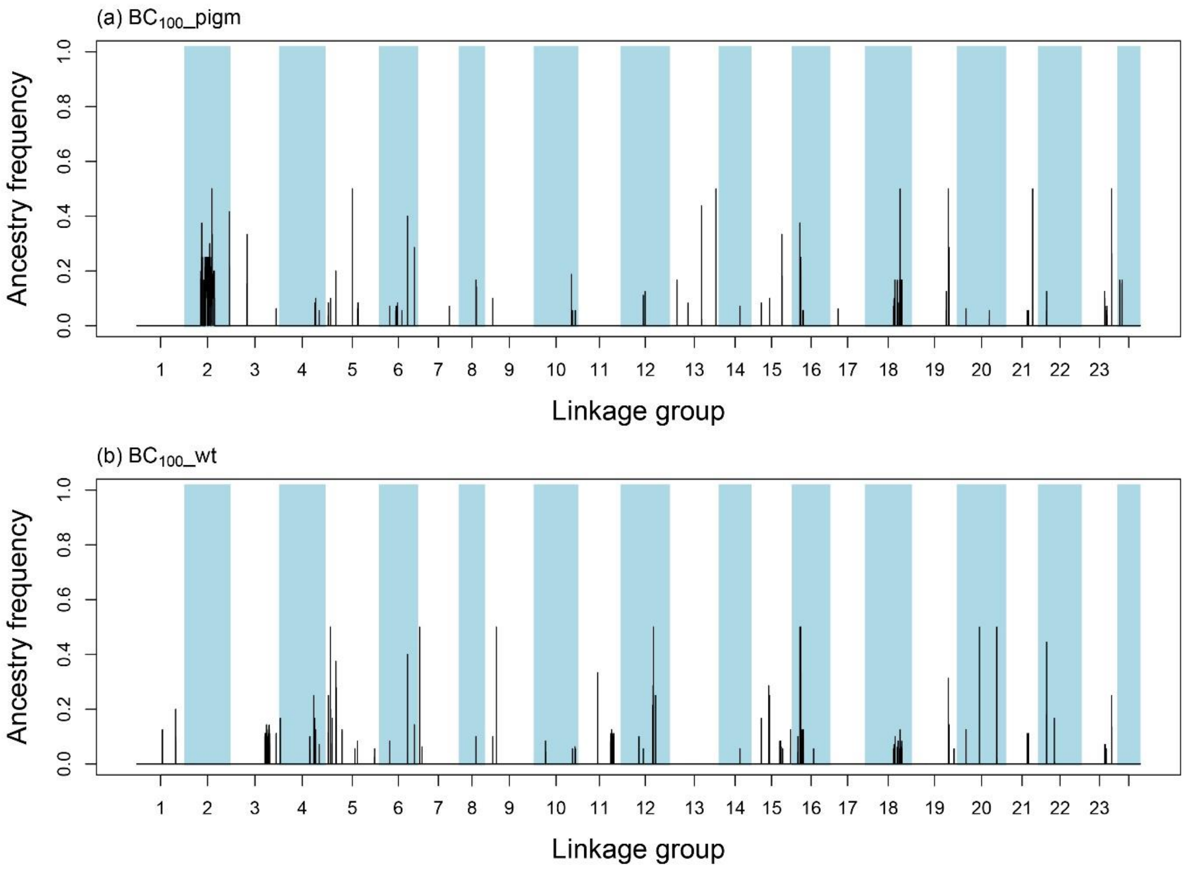

Supplementary Fig. 4. Ancestry frequencies for the BC_{100} lines. Solid lines denote the frequency of platyfish alleles along linkage groups, which are delimited by white and blue background. Null simulations suggest that all ancestry frequencies should be 0 in the absence of selection given the 100 generation backcross experimental design.
