# Supplementary material for: Long-term experimental hybridisation results in the evolution of a new sex chromosome in swordtail fish
Source: Nat Commun. 2018 Dec 3;9:5136. doi: 10.1038/s41467-018-07648-2 (PMC6277394; doi:10.1038/s41467-018-07648-2)
Supplement: Supplementary file 2 — Description of Additional Supplementary Files [file 41467_2018_7648_MOESM2_ESM.pdf]

Description of Additional Supplementary Files

File Name: Supplementary Data 1
Description: Summary statistics of the 60 Xiphophorus hellerii individuals used to characterize the sex determination system of this swordtail species. Sample name, sex (M=male, F=female), pool ID, 8-base i5 and i7 outer barcodes, 6-base i5 and i7 inner barcodes, genetic class, total number of sequences before and after quality filtering, number of RAD loci detected and average coverage are shown for each individual. We obtained a total of 231 million (M) pairedend sequences from one Illumina lane with read length of 151 bp. After the filtering step was applied, we retained 209 M reads (mean: 3.48 M; sd: 0.78 M).

File Name: Supplementary Data 2
Description: Location of the SNPs highly associated with sex in X. hellerii that exceed both the Bonferroni corrected (p-value = 7.64e-7) and the suggestive (p-value = 0.0001) significance thresholds.

File Name: Supplementary Data 3
Description: . For the three sliding window sizes used (1 Kb, 10 Kb and 100 Kb), the genomic regions showing an average FST value in the upper 1% of the distribution of the per window FST values are reported.

File Name: Supplementary Data 4
Description: Summary statistics of the W-linked and Z-linked loci identified by the coverage analysis. The table reports RAD-locus IDs, coverage information for the male and female groups, and matching chromosome and genomic coordinates in both X. hellerii and X. maculatus genomes. The whole set of loci in FASTA format was deposited in the Dryad Digital Repository (doi:10.5061/dryad.7h54h66).

File Name: Supplementary Data 5
Description: Summary statistics of the laboratory cross dataset used. Sample name, 5-base RAD barcode, sex (M=male, F=female), genetic class, total number of sequences before and after quality filtering, number of RAD loci detected and average coverage are shown for each of the 46 individuals. We obtained a total of 125 million (M) single-end sequences from one Illumina lane with read length of 151 bp. After filtering, we retained 105 M reads (mean: 2.28 M; sd: 0.76 M).
